# Nicotine Degradation by *Trametes versicolor*: Insights from Diverse Environmental Stressors and Wastewater Medium

**DOI:** 10.3390/molecules30122658

**Published:** 2025-06-19

**Authors:** Bhautik Dave, Ewa Lobos Moysa, Anna Kuźnik

**Affiliations:** 1 Department of Water and Wastewater Engineering, Silesian University of Technology, 44-100 Gliwice, Poland; ewa.lobos.moysa@polsl.pl; 2 Department of Organic Chemistry, Bioorganic Chemistry and Biotechnology, Silesian University of Technology, 44-100 Gliwice, Poland; anna.kuznik@polsl.pl; 3 Biotechnology Centre, Silesian University of Technology, 44-100 Gliwice, Poland

**Keywords:** nicotine degradation, environmental stressors, wastewater medium, fungal bioremediation, synthetic wastewater, white-rot fungi, biodegradation efficiency, NMR spectroscopy, FTIR analysis

## Abstract

Nicotine, a major alkaloid in tobacco, poses significant environmental risks due to its persistence in wastewater. This study explores the degradation of naturally extracted nicotine from tobacco leaves by the white-rot fungus *Trametes versicolor*, aiming to assess its biodegradation capacity under diverse environmental stressors. Nicotine was extracted using a NaOH–petroleum ether method and confirmed through Fourier-transform infrared (FTIR) and nuclear magnetic resonance (NMR) spectroscopy. Biodegradation experiments were conducted using potato dextrose broth and synthetic wastewater as growth media under varying pH (2.5 and 5.20) and temperatures (25 °C and 37 °C). Fungal growth and nicotine degradation were monitored through biomass quantification and NMR-based analysis. Optimal degradation occurred at 25 °C and pH 5.20, particularly in synthetic wastewater, suggesting enhanced fungal adaptation in complex media. Degradation efficiency ranged from 80% to 99%, with synthetic wastewater outperforming conventional media. Extreme conditions, such as pH 2.5 at 37 °C, significantly hindered fungal growth. These findings demonstrate *T. versicolor*’s potential for effective nicotine removal in wastewater and highlight the environmental parameters critical to its performance. This work supports the development of sustainable fungal-based bioremediation strategies for managing nicotine contamination in aquatic environments.

## 1. Introduction

*Nicotiana tabacum* L. is one of the most widely cultivated crops, historically used for chewing, sniffing, and smoking. Tobacco contains around 4000 chemicals, with 1000 released during smoking [1]. Tobacco smoke comprises approximately 5000 reactive compounds, many of which are toxic and carcinogenic [2,3]. Tobacco leaves themselves contain over 3800 known chemicals, increasing to 5000–6000 upon smoking due to pyrolysis [4,5]. Key chemical classes include alkaloids like nicotine, polyphenols, terpenoids, essential oils, and phytosterols [6,7]. Due to this complexity, efficient compound extraction remains a challenge [8]. Nicotine (3-(1-methyl-2-pyrrolidinyl) pyridine) is the major alkaloid in tobacco, accounting for approximately 90% of its alkaloids and present at levels between 0.3% and 3% in various leaf varieties [1]. It is a water-soluble, hygroscopic, and oily liquid with a boiling point of 246–247 °C [9,10]. It is synthesized in the roots and accumulated in leaves depending on the variety and growing conditions [11,12]. Environmental nicotine contamination arises from tobacco-related industrial waste, leaching, and runoff, contributing to soil and water pollution [13,14].

Detected concentrations include 12.6–947 ng/L in reclaimed water, 9340 ng/L in surface water, and 164 ng/L in groundwater, influenced by flow, dilution, degradation, and seasonal factors [15]. Wastewater treatment plants (WWTPs) can also remove nicotine [16,17]. Nicotine removal was best accomplished by secondary and tertiary treatments [15]. However, these conventional techniques do not completely break down or eliminate nicotine, as evidenced by nicotine and other pollutant residues in the effluent range of ng/L to g/L [18]. Complicating matters further is the rise in the use of natural pollutants like nicotine and caffeine [19]. This emerging category of pollutants has garnered attention due to its increasing prevalence and the environmental concerns it raises. Alarmingly, these substances are often discharged into water bodies without adequate consideration for their ecological impact. Factors such as persistence, bioactivity, and interactions with other chemicals are often overlooked, leaving their potential effects poorly understood [20].

Wastewater treatment plants (WWTPs) are vital for maintaining environmental health by breaking down pollutants in wastewater. In the realm of wastewater treatment, it has become apparent that the predominant method for tackling pollutants is not always through microbial degradation but rather via absorption and sedimentation onto sludge. This observation, backed by research, suggests that high concentrations of pollutants often persist within the effluent even after treatment [21,22,23,24]. Central to this process is the diverse microbial community present within these treatment systems. However, the very nature of WWTPs, where different types of wastewaters converge, leads to a complex mix of microorganisms. This amalgamation fosters intense competition among microbial species for resources like nutrients and space, ultimately resulting in the removal of the majority of organisms. Despite this competitive environment, a fraction of microorganisms manage to persist. However, their survival comes at a cost: they may not fully express their typical functions, such as pollutant degradation, due to the challenging conditions within the WWTP. Consequently, identifying specific pollutant-degrading species becomes a daunting task, and even among the remaining organisms, their ability to efficiently degrade pollutants may be compromised. This poses a significant challenge to the effectiveness of WWTPs in removing pollutants from wastewater. Without a specialized microbial community capable of efficient pollutant degradation, the treatment process becomes less effective. This not only jeopardizes environmental health but also undermines the overall purpose of wastewater treatment. Understanding the dynamics of microbial communities within WWTPs is crucial for optimizing treatment processes and ensuring effective pollutant removal [25]. By unravelling the complexities of microbial interactions and their impact on pollutant degradation, researchers and engineers can develop strategies to enhance the efficiency of wastewater treatment, ultimately safeguarding both environmental and public health [26,27,28,29]. Simply relying on absorption and sedimentation onto sludge may not suffice in effectively addressing the growing threat posed by emerging pollutants. Instead, a concerted effort is required to better understand the behaviour and impact of these substances on the environment, ensuring that our water resources remain clean and safe for all [30,31].

White-rot fungi are pivotal in water pollutant degradation due to their diverse enzymatic toolkit, comprising intracellular enzymes, peroxidases, cytochrome P450, ligninolytic enzymes, and laccases. These fungi catalyze an array of reactions like hydroxylation, oxidation, dehalogenation, deamination, and formylation, exerting significant remediation effects [32]. *Trametes versicolor*, *Trametes hirsute*, and *Phanerochaete chrysosporium* efficiently degraded nicotine in tobacco stalk, reducing its content below 500 mg/kg within 10–15 days. Lignin degradation rates reached 37.70–53.75%, accompanied by hemicellulose (24.28%) and cellulose (28.19%) removal. *Phanerochaete chrysosporium* exhibited the highest ligninolytic enzyme activities, including lignin peroxidase (88.62 U·L^−1^) and manganese peroxidase (100.95 U·L^−1^). GC-MS analysis revealed the production of valuable chemicals and fatty acids alongside nicotine and lignin degradation products [33]. *Aspergillus oryzae* 112822, isolated from tobacco leaves, efficiently degrades nicotine, reaching 2.19 g/L in 40 h with maximum cell growth at 3.6 g/L. Identified intermediates via TLC, MS, NMR, FT-IR, and GC-MS reveal a pathway from nicotine to succinic acid via nornicotine, myosmine, N-methylnicotinamide, and 2,3-dihydroxypyridine. This study provides the first fungal nicotine degradation pathway elucidation [34]. *Acinetobacter* sp. TW bioaugmentation in a bioreactor treating COD (3200 ± 50 mg/L) and nicotine (1.0 ± 0.1 g/L) resulted in stable COD removal (80–90%) and 98% nicotine removal. PCR-DGGE analysis revealed increased bacterial diversity in BA systems. Short-chain AHLs aided strain TW colonization, while long-chain AHLs resisted nicotine toxicity. Reduced ROS, protein carbonyls, and 8-OHdG levels in BA systems indicated strain TW’s role in mitigating nicotine toxicity [35]. Bioaugmentation with *Pseudomonas* sp. HF-1 in SBRs treating tobacco wastewater resulted in 100% nicotine degradation and over 84% COD removal within 12 h, significantly correlating (r = 0.928, *p* < 0.01). PCR-DGGE analysis revealed increased microbial diversity and stability in the BA system. Bioaugmentation minimized nicotine toxicity, reflected in reduced PC and DPC levels. Strain HF-1 influenced the microbial community dynamics, enhancing the treatment efficiency in SBRs [36]. Bioremediation is one of the potential microbe-based approaches for cleaning up contaminated environments [37,38]. Multiple enhancement studies are being conducted in this field, including the production of enzymes [39], metabolic pathways [40], gene expression, etc. [41,42,43,44].

This study aims to explore the extraction of natural nicotine from tobacco leaves and evaluate its concentration, alongside investigating the degradation process facilitated by the fungus *Trametes versicolor* under various environmental stressors. It is hypothesized that tobacco leaves inherently contain nicotine, the primary toxic constituent, and through extraction techniques, the successful isolation and quantification of nicotine can be achieved. *Trametes versicolor*, renowned for its capacity to degrade diverse compounds, is expected to degrade nicotine in natural and synthetic wastewater environments efficiently, adaptable to different media and environmental stressors, likely resulting in varied degradation rates. A comparison between synthetic wastewater and PDB medium is anticipated to reveal differences in nicotine degradation rates, providing insights into the efficacy of synthetic wastewater as a replacement approach. Additionally, analytical techniques such as FT-IR and NMR spectroscopy are employed to identify and confirm the presence of nicotine, with distinct peaks in the spectra facilitating its quantification. Moreover, through NMR analysis, this study aims to determine the optimal degradation conditions for nicotine by *Trametes versicolor*, hypothesizing that factors such as pH (2.5 and 5.20), temperature (25 °C and 37 °C), and nutrient availability influence the degradation process, ultimately identifying conditions conducive to maximum degradation. By systematically investigating these hypotheses, the research endeavours to enhance our understanding of natural nicotine extraction, fungal degradation mechanisms, and the impact of environmental factors on nicotine degradation, thus contributing to the development of effective strategies for mitigating the environmental consequences of nicotine-containing waste streams.

## 2. Results and Discussion

### 2.1. Nicotine Concentration and Determination

The extracted nicotine from tobacco was characterized and quantified using proton nuclear magnetic resonance (^1H NMR) spectroscopy. A known quantity of dimethyl diphenylsilane (DMPS) was added as an internal standard (IS) to each sample before NMR analysis. This method allows for a quantitative comparison based on the integral ratios of distinct proton signals between nicotine and the internal standard.

In quantitative NMR (qNMR), the area under a specific ^1H NMR signal (integral) is directly proportional to the number of nuclei (protons) contributing to that signal. By selecting non-overlapping, characteristic signals of nicotine and the internal standard, the molar ratio of the two compounds in the mixture can be determined.

The internal standard signal from DMPS appeared consistently at 0.55 ppm and is known to represent six protons from two methyl groups (–Si(CH_3_)_2_–). Meanwhile, specific well-resolved nicotine signals (e.g., δ 8.53, δ 8.49, δ 7.70) represent one aromatic proton each, allowing reliable integration.

### 2.2. Mycelium Growth Inhibition, Fungal Resistance to Nicotine Concentrations

**Figure 1:**
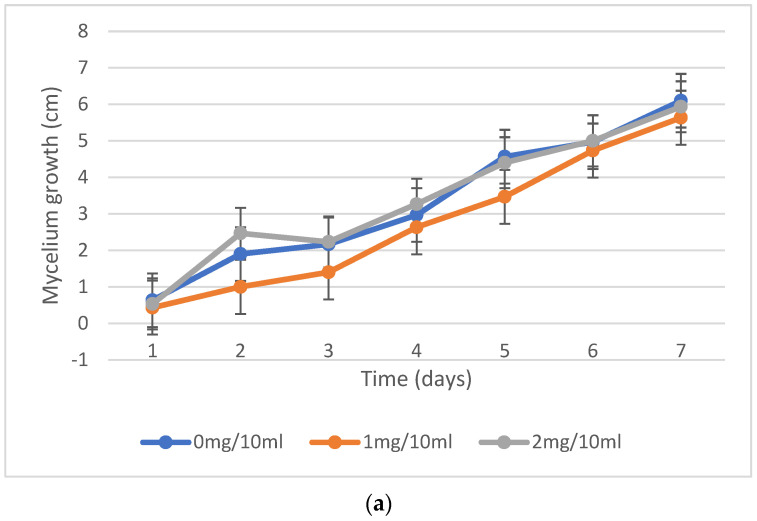

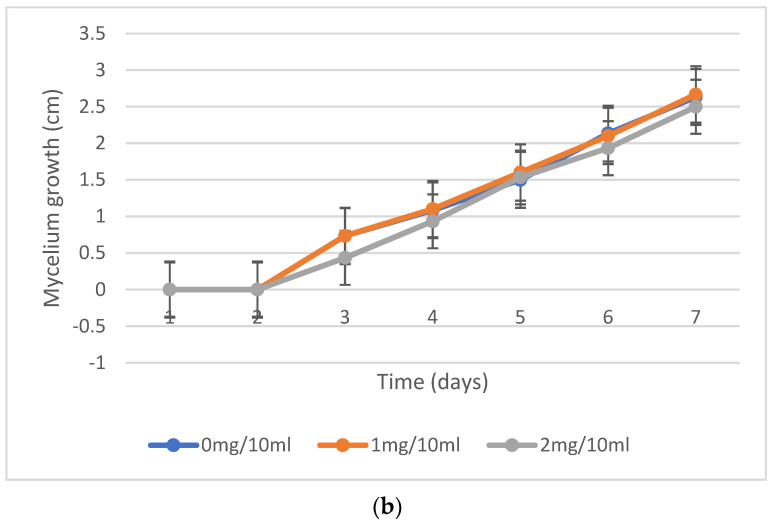
(**a**) Inhibition of fungal growth (incubation at 25 °C; pH of 6.58) with different nicotine concentrations (0, 1, 2 mg/10 mL). (**b**) Inhibition of fungal growth (incubation at 37 °C; pH of 6.58) with different nicotine concentrations (0, 1, 2 mg/10 mL).

**Figure 2:**
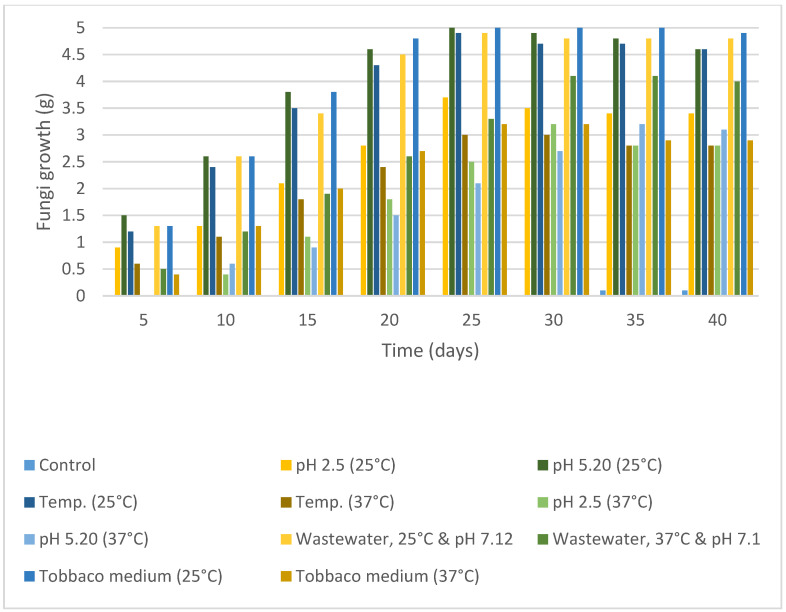
Fungal growth from fungal spores in different samples (liquid media) at a 1 mg/mL nicotine concentration.

In the mycelium growth experiments, there were minor changes in fungal growth between the experimental plates at each concentration at 25 °C incubation (Figure 1a) and the control. However, a prolonged growth rate was observed in plates at 37 °C incubation (Figure 1b). Fungal growth extension on the surface of the medium was very quick. Still, the mycelium growth inside the solid media was much slower, one day behind, on high-concentration plates, specifically with 2 mg/10 mL at both incubations. It turned out that the optimum concentration for performing degradation in liquid media was 1 mg/10 mL. Every five days, the quantification of fungal biomass was performed. The fungal growth and development in every set of flakes with different incubation conditions are shown (Figure 2). From all the samples, synthetic wastewater at 25 °C and the sample at a pH of 5.18 at 25 °C exhibited the fastest growth and high-weight fungal development.

### 2.3. FT-IR Results

**Figure 3:**
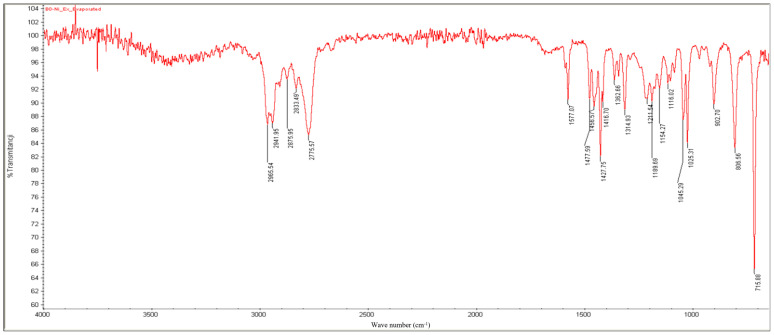
IR spectrum of the nicotine extract obtained.

The following characteristic signals (Figure 3) of the functional groups or other elements of the molecular backbone were detected to confirm the presence of nicotine in the extract obtained:

- Bands between 2966 and 2776 cm^−1^: C-H stretching.
- A slightly split band at 1577 cm^−1^: aromatic C=C and C=N double-bond stretching.
- Bands at 1458, 1428, and 1363 cm^−1^: C-H bending.
- Bands at 903, 806, and 716 cm^−1^: out-of-plane C-H bending of the monosubstituted pyridinic cycle.

### 2.4. NMR Results

**Figure 4:**
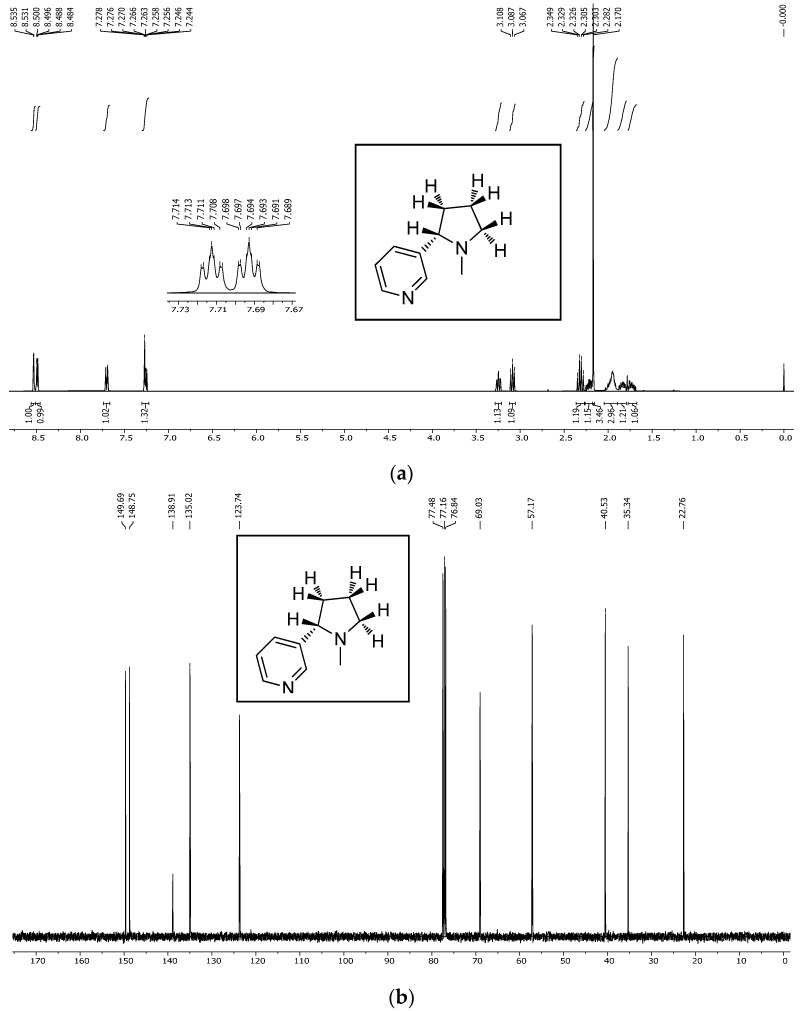
(**a**) 1H NMR spectrum of the nicotine extract obtained. (**b**) ^13^C NMR spectrum of the nicotine extract obtained.

The NMR spectra of the nicotine extract were analyzed using both 1H NMR and 13C NMR techniques, providing detailed insights into the chemical structure of the compound [45,46]. The 1H NMR (Figure 4a) spectrum of nicotine, recorded at 400 MHz in CDCl3, revealed several distinctive chemical shifts and coupling patterns [47]. A broad doublet was observed at δ 8.53 with a coupling constant (*J*) of 1.6 Hz, integrating to one proton. Another signal at δ 8.49 appeared as a doublet of doublets with coupling constants of J1 = 4.8 Hz and J2 = 1.6 Hz, also integrating to one proton. At δ 7.70, a complex doublet of doublets of doublets of doublets (dddd) was noted, with coupling constants of J1 = 8.0 Hz, J2 = 2.2 Hz, J3 = 1.6 Hz, and J4 = 0.4 Hz, integrating to one proton. A doublet of doublets of doublets (ddd) appeared at δ 7.26, with coupling constants of J1 = 7.6 Hz, J2 = 4.8 Hz, and J3 = 0.8 Hz, integrating to one proton. At δ 3.25, another ddd was seen with coupling constants of J1 = 9.6 Hz, J2 = 7.2 Hz, and J3 = 2.2 Hz, integrating to one proton. Additionally, a broad triplet was observed at δ 3.09, with a coupling constant of J = 8.4 Hz, integrating to one proton. Several multiplets were recorded between δ 2.35 and 1.68, integrating to varying numbers of protons: δ 2.35–2.28 (1H), δ 2.24–2.18 (1H), δ 2.03–1.89 (1H), δ 1.87–1.77 (1H), and δ 1.76–1.68 (1H). Lastly, a singlet at δ 2.17 integrated to three protons, indicating the presence of a methyl group [48,49,50].

The ^1H NMR spectrum of the extracted nicotine closely matched the reference (standard) nicotine in terms of the following:

- Chemical shift values;
- Multiplicities;
- Coupling constants;
- Integration values.

This confirmed the following:

- The extracted compound was indeed nicotine;
- It had high purity, as no major impurities or unexpected peaks were observed.

The ^13C NMR further confirmed the identity with carbon shifts matching those reported in the literature and databases (e.g., SDBS), ensuring that the structure remained intact after extraction.

The 13C NMR (Figure 4b) spectrum of nicotine, recorded at 100 MHz in CDCl3, displayed several characteristic chemical shifts. The signals were observed at δ 149.69, δ 148.75, δ 138.91, δ 135.02, δ 123.74, δ 69.03, δ 57.17, δ 40.53, δ 35.34, and δ 22.76. These chemical shifts are consistent with the known carbon skeleton of nicotine, providing further confirmation of the compound’s structure. Overall, the 1H and 13C NMR spectra corroborate the chemical identity of the nicotine extract, with the observed chemical shifts and coupling patterns aligning well with the established structure of nicotine. This analysis confirms the purity and composition of the extracted nicotine.

**Figure 5:**
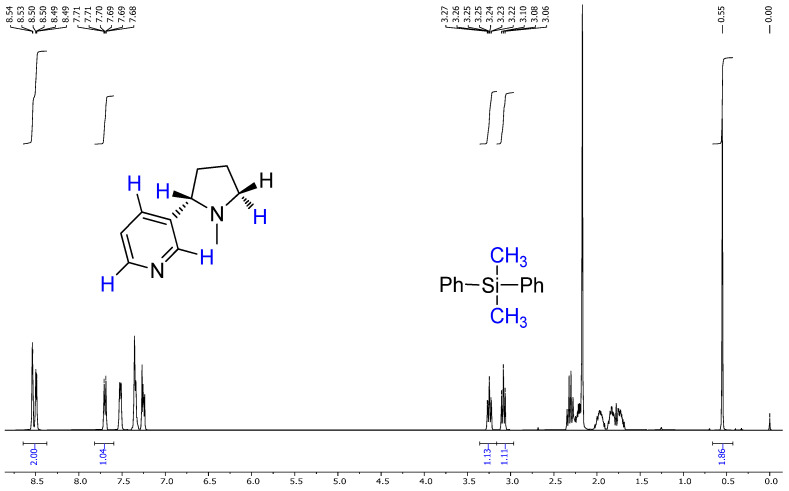
1H NMR spectrum of commercially available nicotine with the addition of a known mass of dimethyldiphenylsilane used as the internal standard.

**Figure 6:**
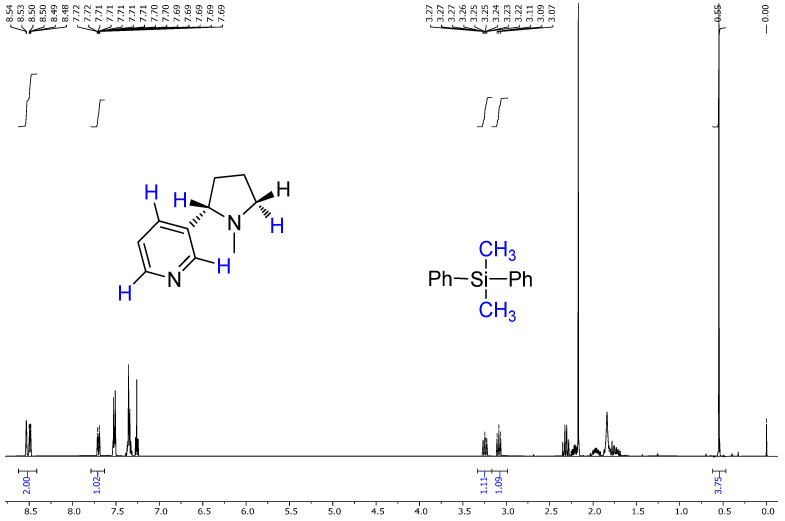
1H NMR spectra of the nicotine extract sample with the addition of a known mass of dimethyldiphenylsilane used as the internal standard.

**Figure 7:**
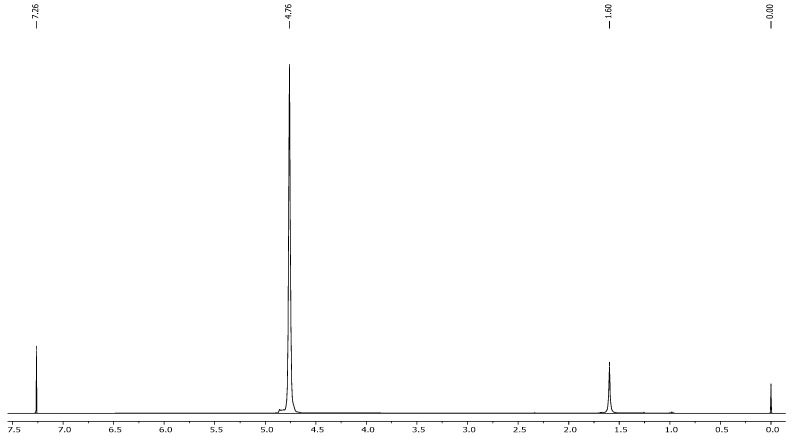
1H NMR spectrum of the nicotine extract after biodegradation (wastewater temperature at 25 °C).

Based on the analysis of the NMR and IR spectra, which are in excellent agreement with the standard reference spectra available in the spectral database (SDBS), the presence of nicotine in the extract was confirmed. The percentage of nicotine in some samples, including the purity of the extract obtained, was determined based on the 1H NMR spectrum relative to the precisely weighed mass of dimethyl diphenylsilane added to the samples as the internal standard. Quantitative calculations consisted of comparing the integration of the average value of the selected nicotine signals with the integration of the internal standard signal appearing at 0.55 ppm (Figure 5 and Figure 6). This comparison considered that the values of integrals per proton of a given compound in the mixture correspond to the molar ratio of these compounds in the analyzed sample. The findings showed that after successful fungal growth at various concentrations and environmental stress conditions, there was faster fungal growth and effective degradation in samples with conditions of 25 °C, pH 5.20 (at 25 °C), and wastewater (at 25 °C) (Figure 7). Other samples showed medium growth and degradation, including samples at 37 °C, pH 2.5 (at 25 °C), pH 5.20 (at 37 °C), wastewater (at 37 °C), and tobacco medium (at 37 °C). The lowest fungal growth was observed in the sample at pH 2.5 and 37 °C, making it an unfavourable condition for fungal growth.

**Figure 8:**
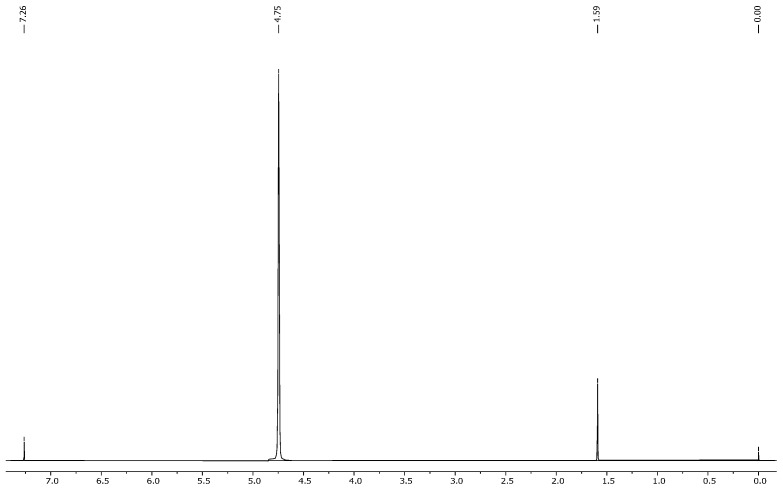
1H NMR spectrum of the tobacco medium; temperature 25 °C.

Fungi proliferated in the tobacco medium at 25 °C (Figure 8) with effective adsorption and degradation. Because of their deficient concentration, it is impossible to estimate the concentration of these traces, which exhibit a degradation of nearly 80 to 99%. Samples with synthetic wastewater showed effective fungi adaptation and growth, indicating the waste medium’s usage for the degradation of specific pollutants through fungal species. All samples with different pH levels showed different fungal growth rates, but all showed positive and effective degradation. By 2 to 3 weeks, the pH of the medium was settled by the enzymes and metabolites produced by the fungi themselves.

### 2.5. Discussion

The investigation into the resistance of fungi to varying concentrations of nicotine, as detailed in the experimental methodology, provides significant insights into fungal adaptation and growth dynamics under diverse conditions. The experiment employed two sets of potato dextrose agar (PDA) plates with distinct nicotine concentrations (1 and 2 mg/10 mL), alongside control plates without nicotine. The method of nicotine application involved uniform distribution across the agar surface. Throughout the seven-day incubation period at both 25 °C and 37 °C, hyphal extension lengths were meticulously measured daily from the colony’s centre to the plate’s border. A quantitative analysis revealed significant differences in fungal growth rates across the various experimental conditions. For instance, at 25 °C, all nicotine concentrations exhibited notable fungal proliferation, with average hyphal extension lengths reaching 1 to 1.5 cm/5 d. In contrast, at 37 °C, growth rates were markedly slower, with average hyphal extension lengths reduced to 0.9 to 1 cm/5 day, indicating a temperature-induced constraint on fungal development.

A further analysis of the data demonstrated intriguing interactions between nicotine concentration, pH, and temperature on fungal growth dynamics. Samples incubated at extreme pH levels (pH 2.5 and pH 5.20) and 37 °C exhibited minimal to no growth, highlighting the combined effect of pH and temperature on fungal adaptation. This observation suggests that extreme pH conditions can impede fungal growth, particularly at higher temperatures, underscoring the importance of considering multiple environmental factors in assessing fungal responses. Moreover, the temporal dynamics of fungal growth revealed distinct patterns across the experimental conditions. Samples incubated at 37 °C lagged approximately 21 days behind those at 25 °C, indicating the temperature-dependent delay in fungal proliferation. Additionally, the cessation of fungal extension from 25 to 45 days, attributed to surface area coverage, underscores the finite growth capacity within the experimental timeframe.

Study from [33] demonstrated that Trametes versicolor, Trametes hirsute, and Phanerochaete chrysosporium are capable of simultaneously degrading nicotine and lignin from tobacco stalks, correlating this activity with the production of high levels of ligninolytic enzymes. Specifically, T. versicolor exhibited the highest laccase activity (745.65 U·L^−1^), T. hirsute showed the highest MnP activity (100.95 U·L^−1^), and P. chrysosporium produced the most LiP (88.62 U·L^−1^). The authors attributed effective nicotine detoxification to the oxidative potential of these enzymes, particularly their ability to oxidize non-phenolic aromatic compounds with the aid of redox mediators.

In addition, study from [51] revealed, through the transcriptome analysis of *Aspergillus oryzae* 112822, that fungal nicotine degradation likely follows a demethylation pathway. They identified upregulated genes encoding cytochrome P450 monooxygenases, FAD-containing amine oxidases, and other oxidative enzymes. The pathway involves initial N-demethylation to nornicotine, followed by dehydrogenation and ring-cleavage reactions, leading to less toxic intermediates like myosmine, nicotinyl alcohol, and nicotinic acid. These findings reinforce that oxidative enzymes, including both ligninolytic and intracellular detoxification enzymes, are integral to fungal nicotine metabolism.

Accordingly, in our study using *Trametes versicolor*, it is highly plausible that similar oxidative mechanisms are at play, especially under the tested conditions that favoured fungal growth and nicotine degradation (e.g., pH 5.2 and 25 °C). We have incorporated this mechanistic insight into the revised discussion to provide context for the observed biodegradation performance, aligning it with existing enzymatic evidence from the literature.

Furthermore, the sustained mycelium growth within the medium beyond the surface extension phase suggests ongoing metabolic activity and nutrient utilization by the fungi. This observation emphasizes the importance of considering surface growth and internal mycelial expansion in assessing fungal behaviour and adaptation. The comprehensive experimental approach, meticulous measurements, and replication elucidate the intricate interplay between nicotine concentration, incubation temperature, and fungal growth dynamics. The findings deepen our understanding of fungal responses to environmental stimuli and underscore the multifaceted nature of fungal adaptation mechanisms. Moreover, statistical analyses were performed to assess the significance of the observed trends, revealing a clear dependence of fungal growth rates on nicotine concentration and temperature. Notably, samples with higher nicotine concentrations exhibited a dose-dependent inhibition of fungal growth, corroborating previous studies on the fungicidal properties of nicotine [52,53,54].

The interpretation of these findings suggests potential mechanisms underlying fungal adaptation to nicotine and environmental stressors. It is plausible that fungi may employ detoxification mechanisms to overcome the inhibitory effects of nicotine, such as enzymatic degradation or efflux pumps [51,55,56]. Additionally, the observed cessation of hyphal extension after a certain period suggests a finite growth capacity within the experimental timeframe, highlighting the need for further investigation into the metabolic dynamics of fungal growth. A comparative analysis with the existing literature on fungal responses to nicotine and environmental stressors reveals both alignment and divergence with previous findings. While our results corroborate the inhibitory effects of nicotine on fungal growth, the observed interactions with pH and temperature provide novel insights into the multifaceted nature of fungal adaptation mechanisms.

The results of the experiments conducted to assess fungal growth and degradation under various environmental conditions provide valuable insights into the adaptability and efficacy of *Trametes versicolor* in biodegradation processes. At an incubation temperature of 25 °C, a sample pH of 5.20 (temperature at 25 °C), and sample wastewater (temperature at 25 °C) (Figure 7) exhibited faster fungal growth and more effective degradation compared to other conditions. It suggests that moderate temperatures and neutral pH levels are conducive to optimal fungal proliferation and degradation activities. Furthermore, synthetic wastewater as a growth medium facilitated effective fungal adaptation and growth, highlighting its potential utility in pollutant degradation through fungal-mediated processes. Other samples have shown medium growth and degradation, including a sample temperature at 37 °C, a sample pH of 2.5 (temperature at 25 °C), a sample pH of 5.20 (temperature at 37 °C), sample wastewater (temperature at 37 °C), and tobacco medium (temperature at 37 °C) [57]. Similarly, [51] offered a transcriptomic perspective on *A. oryzae* 112822, revealing 4381 nicotine-responsive differentially expressed genes. Key enzymes (cytochrome P450s, FAD-, and Moco-containing hydroxylases) were implicated in nicotine demethylation, alongside antioxidant systems (SOD, CAT, Prx) and detoxification-related transporters (ABC and MFS), while growth-related pathways were suppressed, confirming stress adaptation. These findings collectively underscore the biodegradation efficiency and metabolic versatility of fungi in treating nicotine pollution.

In contrast, samples exposed to unfavorable conditions—such as a pH of 2.5 combined with a temperature of 37 °C—showed the least fungal growth, highlighting the detrimental effects of highly acidic environments and elevated temperatures on fungal development. Interestingly, despite being exposed to suboptimal conditions, samples containing tobacco medium (temperature at 25 °C) (Figure 8) demonstrated the effective adsorption and degradation of pollutants. This underscores the adaptability of *Trametes versicolor* to utilize different growth media for efficient pollutant degradation. The degradation efficiency of pollutants, estimated to be nearly 80% to 99%, highlights the effectiveness of *Trametes versicolor* in removing contaminants from the medium. However, it is worth noting that the precise estimation of pollutant concentrations is challenging due to their low concentration. Nonetheless, the observed degradation rates underscore the potential of *Trametes versicolor* as a promising candidate for pollutant remediation applications (Table S1). The degraded products are not identified, but it is an important part in this kind of research because the end products or even the intermediates can have toxicity, and we need to consider their environmental safety [58]. For this, the whole degradation pathway and fungal metabolism should be characterised. Each intermediate and metabolite should be identified by a proper analytical technique [59,60].

The study from [34] identified *Aspergillus oryzae* 112822 from tobacco leaves and proposed a novel fungal nicotine degradation pathway involving intermediates, such as nornicotine, myosmine, and 2,3-dihydroxypyridine, ultimately leading to succinic acid. After 40 h of cultivation in tobacco leaf extract, the fungus achieved a maximum biomass of 3.6 g/L and degraded 2.19 g/L of nicotine. Expanding the practical application [33] demonstrated the potential of white-rot fungi (*Trametes versicolor*, *Trametes hirsute*, and *Phanerochaete chrysosporium*) to simultaneously degrade nicotine and lignin in tobacco stalks. After fermentation, nicotine levels dropped to below 500 mg/kg, a safe environmental threshold, while lignin degradation reached up to 53.75%. Valuable enzymes (e.g., laccase at 745.65 U·L^−1^) and small molecules, including fatty acids and other by-products, were identified, indicating biotechnological potential in waste valorisation.

The variations in fungal growth rates observed among samples with different pH levels underscore the significant role of environmental factors in shaping fungal metabolism and adaptation mechanisms. This underscores the self-regulatory capabilities of *Trametes versicolor* in modifying its surroundings for optimal growth and degradation, as pH levels settle over time due to enzymatic activities and metabolite production by the fungi. Moreover, the utilization of synthetic wastewater as a growth medium yielded promising results, demonstrating its potential to facilitate the degradation of specific pollutants by fungal species. Samples with synthetic wastewater showed effective fungal adaptation and growth, suggesting the potential utilization of waste media for biodegradation processes. The observed variation in fungal growth rates across different pH levels emphasizes the importance of environmental factors in fungal biodegradation processes. Additionally, the ability of fungi to adapt and thrive in various conditions highlights their potential as valuable agents for bioremediation efforts.

Furthermore, these degrading technologies are used in practical applications and waste environments and have shown that aerobic composting may remove 80% of nicotine and 50% of the volume and mass of tobacco solid waste in 16 days. Various nicotine-degrading bacteria and fungi are used in the biological technique [61]. Because of their high effectiveness and low cost, these environmentally friendly physical technologies are widely utilized in wastewater treatment. Microbes that break down nicotine have been shown to adapt to contaminated environments quickly [62,63]. Nicotine can be degraded by native strains of bacteria and fungi found in the tobacco environment [64,65]. Fungal species, including *Pellicularia filamentosa*, *Cunninghamella echinulate*, and *Aspergillus oryzae*, have proven to be effective nicotine-degrading fungi. *Pleurotus filamentosa* JTS-208 degraded 0.04 g/l of nicotine in 20 days and accumulated only one metabolite, nicotine, while *Cunninghamella echinulate* IFO-4444 degraded 0.54 g/L of nicotine in 13 days and accumulated nicotine, N-methylmyosmine, and three unidentified metabolites in the medium [66]. *Aspergillus oryzae* 112822, a nicotine-degrading fungus isolated from tobacco leaves, was recently found. For 40 h, it was cultivated in a medium containing tobacco leaf extract. The most significant amount of cell growth was 3.6 g/L, whereas the maximum quantity of nicotine breakdown was 2.19 g/L [34].

These findings have significant implications for future environmental remediation strategies because we have degraded the selected pollutant in potential fungal media and also in synthetic wastewater medium, which is novel in terms of the microbiological perspective. We have proved the efficiency of fungal degradation inside the waste medium and compared it with the ideal PDB medium. The high efficacy of fungal degradation observed in this study suggests the feasibility of utilizing fungi for the removal of nicotine and other pollutants from contaminated environments. By harnessing the natural biodegradative capabilities of fungi, cost-effective and environmentally friendly approaches for the remediation of polluted sites can be developed. Furthermore, the utilization of synthetic wastewater as a growth medium presents a sustainable solution for enhancing fungal growth and degradation performance [67,68,69]. In conclusion, this study demonstrates the promising potential of fungal biodegradation for mitigating nicotine contamination and environmental pollution. The further research and implementation of bioremediation strategies utilizing fungi in real waste medium could significantly contribute to environmental conservation and public health efforts.

## 3. Materials and Methods

### 3.1. Chemicals and Materials

Pure raw tobacco leaves were purchased from the local Polish market. These tobacco plants are without any pretreatment or processing. Sodium hydroxide (NaOH—99%), petroleum ether (98%), anhydrous potassium carbonate (99%), sulphuric acid (99%) and syringe filters, nicotine standard, dimethyldiphenyl silane, and deuterated chloroform were purchased from Merck, Poland. The microbial medium, potato dextrose agar, was purchased from HiMedia, Mumbai, India.

### 3.2. Nicotine Extraction

The extraction of nicotine entirely depends on isolating the base by dissolving the tobacco in NaOH; so, in the first step, 10 g of tobacco was mixed in 150 mL of 40% NaOH solution. This solution was adequately mixed and then put on the mechanical shaker at 250 rpm for one hour. A slower but efficient rotation was required because the tobacco leaves should absorb the NaOH solution. After that, a very slow mixture was given to the solution using the magnetic stirrer and hot plate (50 °C) until a strong smell of tobacco emerged. High temperature threatens nicotine, which will hydrolyse by extreme or direct high heating. After that, the whole solution was filtered through the filter paper.

Nicotine is miscible in water at 60 °C, around 1000 mg/mL. It dissolves with multiple compounds, including alcohol, ether, and petroleum ether [70]. Various solvents are investigated for nicotine extraction from tobacco leaves; each solvent gives different rates of extracted nicotine. Petroleum ether, isooctane, benzene, and chloroform are the most widely used [71]. After successful extraction, purification is also necessary for pure nicotine.

For nicotine extraction from the filtered solution, 30 mL of petroleum ether was added and covered with aluminum foil to stop the evaporation of the ether. The solution was mixed for 15 min on a magnetic stirrer. After that, the whole solution was transferred into the large 1-litre separating funnel, and both layers were separated and collected in different beakers. The large surface area of the separating funnel increased the extraction yield. The beaker with the extracted bottom layer was stored, and the whole extraction was repeated with the top layer. It directly increased the yield of the nicotine. As mentioned before, the nicotine yield can be affected by heavy segregates and bubble formation. By repeating the extraction step, the increment will be possible. [4] After repetition, the whole extracted bottom layer was collected in a beaker. This extract still contained a lower amount of ether, so it was evaporated in a water bath at a temperature of 45 °C until the smell of the ether stopped. All ether evaporated after 10 to 15 min, and oily yellow-brownish particles and droplets were visible. This method is considered the most cost-effective method, specifically for nicotine extraction.

### 3.3. Nicotine Concentration and Determination

Nicotine concentration and determination were made by FT-IR and NMR analysis. The pure nicotine as a reference sample (Sigma Aldrich, Poznań, Poland) and the extracted nicotine were compared and determined by adding the exact mass of the internal standard (dimethyl diphenyl silane).

### 3.4. Cultural Condition of Trametes Versicolor

Identified species of *Trametes versicolor* were collected from the Department of Air Protection, Silesian University of Technology, Gliwice, Poland [72], and were utilized for fungal degradation applications. The cultivation procedure entailed using the dilution–plating method on potato dextrose agar plates. These plates were then subjected to a seven-day incubation at 25 °C to foster ideal fungal proliferation and adaptation.

### 3.5. Mycelium Growth Inhibition by Different Nicotine Concentrations

The resistance of fungi to various concentrations of nicotine from the extract was investigated. Two sets of PDA plates were made with 1 and 2 mg/10 mL concentrations, while plates without nicotine were used as controls. Every day, the lengths of the hyphal extensions of each fungus were measured from the colony’s centre to the plate’s border while incubating at 25 °C and 37 °C, respectively, for 7 days. This experiment was conducted in triplicate [73].

### 3.6. Fungi Biomass Quantification

It was essential to assess the selected fungus’s capacity for adaptability, the rate of development under various stress situations, and how well it can use the medium. The weight of each flask was measured every five days to quantify the fungal biomass. The total weights of the flask and medium before and after the fungal inoculation were compared every five days during the experiment.

### 3.7. Biodegradation Experiments

The experiment was meticulously crafted to investigate the biodegradation capabilities of a selected fungal strain across varying environmental conditions while maintaining a consistent nicotine concentration of 1 mg/10 mL throughout all experimental setups. To explore the impact of temperature fluctuations, two sets of flasks, each containing 100 mL of potato dextrose broth (PDB) media, were utilized. One set was kept at 25 °C while the other was maintained at 37 °C to unravel the temperature’s influence on biodegradation efficiency. Similarly, to examine the effects of pH variations, two sets of flasks containing 100 mL of PDB media were employed. One set maintained a standard pH of 5.18 (considered normal), while the pH of the other set was adjusted to 2.5 by the addition of hydrochloric acid, allowing for an exploration of the pH’s impact on biodegradation efficiency (Table 1).

An additional set of flasks was filled with synthetic wastewater, following a specific composition, which included Peptone: 0.16 g/L, broth: 0.11 g/L, Urea: 0.03 g/L, K_2_HPO_4_: 0.028 g/L, NaCl: 0.007 g/L, CaCl_2_∙2H_2_O: 0.004 g/L, and MgSO_4_∙7H_2_O: 0.002 g/L with a pH of 6.4 outlined by [74]. In each flask, a concentration of 1 mg/10 mL of nicotine was introduced, and thorough agitation ensured proper mixing. Moreover, one set of flasks was augmented by adding 15 g of tobacco to 100 mL of synthetic wastewater medium. Control flasks were established using MilliQ water as the medium. Subsequently, these flasks were subjected to two different incubation temperatures, 25 °C and 37 °C, facilitating the observation of potential temperature-dependent effects on fungal degradation processes.

Following this, a precise 1 mL portion of medium containing fungal spores was carefully inoculated into each flask. The inoculation process was conducted meticulously, involving the extraction of a 1 mL sample, followed by vigorous blending on a vortex within a sterile environment. This ensured the homogeneous distribution of fungal spores and mycelia throughout the medium. The abundance of fungal spores introduced during inoculation serves to bolster the likelihood of fungal survival and its ability to withstand potential toxins. This enriched mixture also fosters the establishment of a robust network of fungal growth, which blankets the entire surface of the flask and permeates the medium. As a result, rapid fungal proliferation can be observed within a relatively short span of 4 to 7 days, particularly evident in liquid medium configurations. Such a meticulously crafted experimental setup offers a comprehensive platform for delving into the degradation processes of nicotine and tobacco leaves by the *Trametes versicolor* fungus under diverse environmental conditions.

### 3.8. Sample Extraction and Purification

After extensive incubation and significant fungal growth in the medium, removing and isolating the degraded product is essential. Numerous techniques for eliminating microbes were developed, most of which relied on membrane filtration, absorption, and separation. In an aseptic environment, 50 mL of samples was obtained and placed in a sterile centrifuge tube using a sterile injection and a medical syringe. The bulk of large biomass and fungal growth can be prevented, allowing the collection of only liquid samples. All the tubes are centrifuged at 7000 rpm for 20 min in the second stage for the settlement of the solid particles present in the samples. Through another sterile injection and medical syringe, the supernatant was extracted. In the third phase, every sample was run through nylon syringe filters (0.45 mm and 4 mm). The most crucial step is syringe filtration, which uses sterile injections coupled to syringe filters at the end with thumb pressure. Around 1 to 2 mL of fluid can be filtered from each syringe. Each syringe filtration yielded 2 to 4 mL of samples, which were then collected in a sterile glass bottle. The collected sample was kept in a hot-air oven for an hour at 40 °C to allow the water to evaporate. Each sample was prepared for NMR examination. [73,75].

### 3.9. FT-IR Analysis

Nicotine was collected and measured on an FT-IR instrument (Thermo Scientific—Nicolet 6700, Waltham, MA, USA, attenuated total reflectance method; ATR). A total of 2 mg of extracted solid powder nicotine was measured in FT-IR. This nicotine solid powder was prepared after 24 h of water evaporation at 40 °C in a hot-air oven. The FT-IR spectrum of extracted nicotine is illustrated in Figure 3.

### 3.10. NMR Analysis

1H NMR and ^13^C NMR spectra were recorded on a Varian 400 spectrometer at 400 MHz and 100 MHz frequencies. 1H NMR chemical shifts are reported relative to tetramethylsilane (TMS) as the internal standard. ^13^C NMR chemical shifts are reported from the solvent resonance employed as the internal standard (CDCl_3_ at 77.16 ppm). All chemical shifts (δ) are reported in ppm and coupling constants (*J*) in Hz.

## 4. Conclusions

In conclusion, our research sheds light on the adaptability and effectiveness of *Trametes versicolor* in biodegrading nicotine, offering valuable insights for sustainable bioremediation practices. By employing robust analytical techniques like FTIR and 1H NMR spectroscopy and meticulous experimental methodologies, we successfully extracted and characterized nicotine from samples. Our experiments also revealed the fungus’s resistance to nicotine concentrations and environmental stressors, highlighting the intricate interplay between environmental factors and fungal growth dynamics. Our findings underscore *Trametes versicolor*’s remarkable ability to degrade nicotine, offering promising avenues for sustainable bioremediation strategies. Optimizing fungal-based approaches for practical applications is essential to advance environmental science and promote ecosystem restoration. Leveraging wastewater as a nutrient source for microbial biodegradation can efficiently scale up remediation efforts, addressing various pollutants in diverse ecological settings. Despite challenges posed by microbial diversity, this interdisciplinary approach holds significant potential in mitigating environmental pollution and safeguarding ecosystem health for future generations.

Future research should focus on elucidating the mechanisms underlying fungal adaptation and degradation processes, including identifying key enzymes and metabolic pathways involved in nicotine degradation. Exploring the synergistic effects of microbial consortia could enhance degradation efficiency and broaden the applicability of fungal-mediated approaches in environmental clean-up efforts. Moreover, investigating the scalability and feasibility of implementing fungal-based bioremediation technologies in real-world contaminated environments is crucial.

## Figures and Tables

**Table 1:** Experimental samples with conditions.

No.	Fungi Inoculation	Concentration of Nicotine	Incubation
1.	Temperature at 25 °C (PDB)	1 mg/10 mL	25 °C
2.	Temperature at 37 °C (PDB)	1 mg/10 mL	37 °C
3.	pH 2.5 (25°) (PDB)	1 mg/10 mL	25 °C
4.	pH 2.5 (37°) (PDB)	1 mg/10 mL	37 °C
5.	pH 5.18 (25°) (PDB)	1 mg/10 mL	25 °C
6.	pH 5.18 (37°) (PDB)	1 mg/10 mL	37 °C
7.	pH 6.4 (25°) (SWW)	1 mg/10 mL	25 °C
8.	pH 6.4 (37°) (SWW)	1 mg/10 mL	37 °C
9.	15 gm of tobacco (100 mL of SWW)	-	25 °C
9.	15 gm of tobacco (100 mL of SWW)	-	37 °C
10.	Control sample (MilliQ water 25°)	1 mg/10 mL	25° C
11.	Control sample (MilliQ water 37°)	1 mg/10 mL	37° C

## Data Availability

All data supporting the findings of this study are available within the paper and its Supplementary Information.

## Supplementary Materials

The following supporting information can be downloaded at: https://www.mdpi.com/article/10.3390/molecules30122658/s1, Table S1: Experimental parameters and results.
