# Supplementary material for: Nicotine Degradation by Trametes versicolor: Insights from Diverse Environmental Stressors and Wastewater Medium
Source: Molecules. 2025 Jun 19;30(12):2658. doi: 10.3390/molecules30122658 (PMC12196271; doi:10.3390/molecules30122658)
Supplement: Supplementary file 1 [file molecules-30-02658-s001.zip › molecules-3702983-supplementary.pdf]

Nicotine Degradation by Trametes versicolor: Insights from Diverse Environmental Stressors and Wastewater Medium

Bhautik Dave 1,*, Ewa Lobos Moysa 1 and Anna Kuźnik 2,3

1 Department of Water and Wastewater Engineering, Silesian University of Technology, 44-100 Gliwice, Poland
2 Department of Organic Chemistry, Bioorganic Chemistry and Biotechnology, Silesian University of Technology, 44-100 Gliwice, Poland
3 Biotechnology Centre, Silesian University of Technology, 44-100 Gliwice, Poland
* Correspondence: bhautik.dave@polsl.pl or bhautikdave1997@gmail.com

Table. S1 Experimental parameters and results

**Sample**	**Condition**	**Concentration**	**Fungal biomass**	**NMR Results***	**Degradation**	**Result**
**1**	Control 25°C& 37°C	1mg/10ml	-	32.56	-	-
**2**	Sample, Temp. 25°C	1mg/10ml	4.6g	0.62	High growth and degradation, 98%	+, +, +, +
**3**	Sample, Temp. 37°C	1mg/10ml	2.8g	2.31	Low growth and absorption, 92%	+
**4**	Sample, pH 2.5 (Temp. 25°C)	1mg/10ml	3.4g	2.42	Slow growth and degradation, 92%	+, -
**5**	Sample, pH 5.20, (Temp. 25°C)	1mg/10ml	4.6g	0.61	High growth and Degradation, 98%	+, +, +, +

**6**	Sample, pH 2.5, (Temp. 37°C)	1mg/10ml	2.8g	1.58	Slow growth and absorption, 95%	-, -
**7**	Sample, pH 5.20, (Temp. 37°C)	1mg/10ml	3.1g	1.47	Medium growth and degradation, 95%	+, -
**8**	Sample, wastewater Temp 25°C, pH 7.1	1mg/10ml	4.8g	0.63	High growth and degradation, 98%	+, +, +,+
**9**	Sample, wastewater Temp 37°C, pH 7.1	1mg/10ml	4.0g	1.36	Medium growth and degradation, 95%	+, +
**10**	Tobacco medium, Temp 25°C,	-	4.9g	-	High growth and degradation	+, +
**11**	Tobacco medium, Temp 37°C,	-	2.9g	-	Medium growth and absorption	+

* - Comparison of caffeine signal integration in samples before biodegradation (entry 1) and after biodegradation (entries 2 - 11).

Semi-Quantitative Estimation of Nicotine Concentration

- W_IS (mass of internal standard) = 1.00 mg
- MW_IS (molecular weight of DMPS) = ~198.39 g/mol
- MW_Nic (molecular weight of nicotine) = 162.23 g/mol
- From NMR, the integral of IS at 0.55 ppm = 6.0 (representing 6 protons)
- A selected nicotine signal (e.g., δ 8.49) has an integral = 1.0 (for 1 proton)

Then, molar amount of IS in sample:

$nIS=1.00\ mg\div198.39\ g/mol=5.04\times10-6\ mol$

From the ratio of integrals:

$Integralnic/Hnic\div IntegralIS/HIS=1/1\div6/6=1\Rightarrow nnic=nIS=5.04\times10-6\ mol$

Mass of nicotine:

$mnic=nnic\times MWnic=5.04\times10-6\times162.23\approx0.818$

If this mass of nicotine is dissolved in 10 mL solution:

$Concentration=0.818\ mg\div10\ mL=0.0818\ mg/mL$

If integrals slightly differed (e.g., nicotine integral was 1.2), the calculated nicotine mass would be:

$1.26\div6\div6\div6=1.2\Rightarrow nnic=1.2\times nIS=6.05\times10-6\ mol\Rightarrow mnic=6.05\times10-6\times162.23\approx0.982\ mg\Rightarrow0.982\div10\ mL\approx0.0982\ mg/mL$

Thus, a measured concentration of ~1 mg per 10 mL can be reliably estimated through careful signal integration and known mass of internal standard.

Quantification of Nicotine Degradation via NMR

The extent of nicotine degradation was semi-quantitatively estimated by comparing the integrated peak areas of the nicotine signals in the ^{1}H NMR spectrum of the degraded sample to that of a standard (undegraded) nicotine reference.

The calculation follows this formula:

Degradation Ratio=NMR integral of standard nicotine ÷
NMR integral of degraded sample nicotine peak

This ratio reflects how much nicotine remains after fungal treatment compared to the original (undegraded) sample. From this, we calculate:

- Percentage Degraded:

Percentage Degraded=(NMR integral of degraded nicotine÷NMR integral of standard nicotine)×100%

- Remaining (Non-degraded) Nicotine:

Remaining Nicotine (%)=(NMR integral of degraded nicotine÷NMR integral of standard nico tine)×100%
